# Comprehensive Cardiac Rehabilitation in Heart Failure: A Narrative Review of Phenotype-Specific Modalities, Risk Stratification, and Clinical Outcomes

**DOI:** 10.3390/healthcare14213592

**Published:** 2026-10-23

**Authors:** Kamil Salwa, Karol Kaziród-Wolski, Magdalena Wolska, Dorota Rębak, Janusz Sielski

**Affiliations:** 1 Intensive Cardiac Care Unit, Świętokrzyskie Cardiology Center, 25-736 Kielce, Poland; karol.kazirod-wolski@ujk.edu.pl (K.K.-W.); jsielski7@interia.pl (J.S.); 2 Institute of Health Sciences, Collegium Medicum, Jan Kochanowski University in Kielce, 25-317 Kielce, Poland; dorota.rebak@ujk.edu.pl; 3 Institute of Medical Sciences, Collegium Medicum, Jan Kochanowski University in Kielce, 25-317 Kielce, Poland; mag.wolska@poczta.onet.pl; 4 Outpatient Treatment Facility “CenterMed”, 25-314 Kielce, Poland

**Keywords:** cardiac rehabilitation, heart failure phenotypes, cardiopulmonary exercise testing, exercise prescription, telerehabilitation, precision medicine, peripheral myopathy

## Abstract

**Highlights:**

**What are the main findings?**

• Exercise intolerance in heart failure reflects heterogeneous central and peripheral mechanisms that require individualized assessment.
• Rehabilitation improves functional outcomes and health-related quality of life; effects on hospitalization and mortality should be interpreted separately.

**What are the implications of the main findings?**

• Exercise prescription and supervision should reflect clinical stability, measured responses, comorbidities, and patient preferences.
• Home-based and hybrid delivery offer additional options, while omics-guided rehabilitation remains an emerging research approach.

**Abstract:**

Background/Objectives: Cardiac rehabilitation addresses exercise intolerance and impaired quality of life in heart failure. This review examines clinical assessment, exercise prescription, multidisciplinary care, and rehabilitation delivery across populations described using historical HFrEF, HFmrEF, and HFpEF. Methods: This narrative review discusses randomized trials, observational studies, systematic reviews, professional guidelines, and position statements. Functional and patient-reported outcomes are considered separately from hospitalization and mortality, with attention to population differences and limitations of extrapolation. Results: Exercise intolerance reflects varying contributions from central haemodynamic limitations, peripheral abnormalities, and comorbidities. CPET can support functional assessment and individualized exercise prescription. Aerobic and resistance training improve selected functional outcomes, while complementary interventions should address individual clinical needs. Evidence supports improvements in health-related quality of life and reductions in all-cause hospital admissions, whereas mortality effects remain uncertain. Direct evidence is uneven across historical LVEF categories and advanced HF populations. Conclusions: Rehabilitation should be individualized according to stability, functional capacity, comorbidities, and patient preferences. Historical categories are retained to preserve the population definitions of the underlying studies despite the revised 2026 ESC classification. Home-based and hybrid programmes provide additional delivery options, while omics-guided approaches require further validation.

## 1. Introduction

Heart failure (HF) imposes a substantial global burden of morbidity, mortality, and healthcare use [1,2]. Its prevalence increases with age, while prognosis varies with the population, disease severity, comorbidities, and treatment [3]. Comprehensive care therefore combines guideline-directed medical therapy (GDMT) with rehabilitation addressing exercise intolerance, physical function, and health-related quality of life [4].

Exercise training is an established component of heart failure management in appropriately selected patients. Its benefits include improvements in exercise capacity, skeletal muscle function, and health-related quality of life, although the magnitude and mechanisms of benefit vary between populations and programmes [4,5,6,7,8]. Evidence concerning hospitalization and mortality requires separate interpretation and should not be inferred directly from physiological adaptations.

Comprehensive cardiac rehabilitation combines individualized exercise training with clinical assessment, education, nutritional support, and psychosocial care [4,8]. CPET can support functional assessment and exercise prescription, while programme delivery should account for clinical stability, comorbidities, patient preferences, and available resources [9]. In HFpEF, trials support functional benefits, but improvements in exercise capacity do not necessarily coincide with changes in resting diastolic function; OptimEx-Clin did not establish superiority of HIIT over MICT [10].

This narrative review addresses mechanisms of exercise intolerance, assessment, exercise prescription, multidisciplinary care, and centre-based or remote rehabilitation across heart failure populations. Evidence is organized using the historical categories of HFrEF, HFmrEF, and HFpEF to preserve the population definitions used in the studies discussed [7,11,12]. Among patients with established HF, the 2026 ESC guidelines distinguish HFrEF with LVEF < 50% and HFpEF with LVEF ≥ 50% [13]. Retaining the earlier categories is an organizational choice for evidence interpretation rather than a proposal to retain the superseded ESC classification. Results are interpreted within the populations actually studied, without assuming that findings from one historical subgroup apply uniformly throughout a contemporary LVEF category.

## 2. Materials and Methods

This narrative review examines cardiac rehabilitation in adults with heart failure, focusing on exercise intolerance, clinical assessment, exercise prescription, multidisciplinary care, and rehabilitation delivery. Studies were identified through searches of PubMed/MEDLINE, Scopus, and Google Scholar using terms related to heart failure, cardiac rehabilitation, exercise training, and exercise prescription. The discussion draws on randomized trials, observational studies, systematic reviews, professional guidelines, and position statements. Recent publications are considered alongside earlier foundational studies relevant to contemporary rehabilitation practice. Pharmacological studies and diagnostic commentaries provide clinical context and are distinguished from studies directly evaluating rehabilitation.

Evidence is discussed qualitatively with attention to the population studied, intervention, comparator, outcomes, and follow-up. Functional capacity and health-related quality of life are considered separately from hospitalization and mortality. Guideline recommendations are distinguished from findings of individual studies, mechanistic explanations, and extrapolation to populations with limited direct evidence. This is a narrative synthesis. No new meta-analysis or formal certainty-of-evidence assessment was performed; published pooled estimates are identified and reported as such. Generative AI tools were used to assist manuscript minor revisions. Proposed changes and source attributions were strictly reviewed by all of the authors.

## 3. Results

### 3.1. Pathophysiology of Exercise Intolerance in Heart Failure

#### 3.1.1. Central Hemodynamic Impairments

According to the Fick principle, oxygen uptake reflects cardiac output and the arteriovenous oxygen-content difference. In HF, impaired augmentation of cardiac output may contribute to exercise limitation, but the relative contributions of central and peripheral abnormalities vary between patients [4]. HFpEF is heterogeneous: impaired relaxation, increased ventricular stiffness, limited stroke-volume reserve, chronotropic impairment, and peripheral abnormalities contribute to exercise intolerance to varying degrees [14,15]. Filling pressures may rise predominantly during exercise despite inconclusive resting findings; exertional dyspnea with preserved LVEF does not by itself establish HFpEF [16]. Chronic exercise limitation should therefore be distinguished from overt congestion or decompensation. Chronotropic responses should be interpreted in relation to medication, rhythm, and achieved effort. Right ventricular–pulmonary vascular dysfunction can provide an additional limitation in selected patients [14].

#### 3.1.2. Peripheral Alterations

Peripheral contributors to exercise intolerance include skeletal muscle weakness, reduced oxidative capacity, and impaired oxygen delivery or utilization [4,5]. Their relative importance varies between patients and interacts with central hemodynamics, comorbidities, body composition, and physical deconditioning. These abnormalities can contribute to functional limitation without being the dominant mechanism in every patient.

#### 3.1.3. Ventilatory Inefficiency

Ventilatory inefficiency in HF may reflect altered pulmonary perfusion, increased dead-space ventilation, enhanced ventilatory drive, and abnormal muscle-reflex responses [4,5]. An elevated VE/VCO_2_ slope provides prognostic information, but interpretation depends on the population, clinical context, and test methodology [4]. The ergoreflex comprises mechanoreflex and metaboreflex components, with signals conveyed predominantly by thinly myelinated group III and unmyelinated group IV muscle afferents [5]. Altered reflex sensitivity may contribute to excessive sympathetic and ventilatory responses [5]. Pulmonary congestion and inspiratory muscle weakness can contribute in selected patients; chronic exertional dyspnea should not automatically be attributed to acute pulmonary edema [4].

### 3.2. Clinical Assessment and Risk Stratification Prior to Rehabilitation

#### 3.2.1. Baseline Clinical Evaluation, Echocardiography, and Biomarker Profiling

Before rehabilitation, clinical assessment should establish current stability, congestion, orthostatic symptoms, comorbidities, and medication tolerance [4,8]. Rehabilitation may begin after sufficient stabilization, with timing determined by clinical status and the proposed intervention rather than a fixed waiting period [13,17]. Echocardiography assesses ventricular and valvular structure and function, whereas E/e′ contributes to a multiparametric estimate of filling pressure rather than providing a direct measurement [16]. BNP and NT-proBNP should be interpreted alongside clinical findings, age, rhythm, obesity, and renal function. An unexpected increase warrants reassessment for decompensation or other causes, but an isolated value or trend does not independently determine exercise eligibility. Renal function, electrolytes, haemoglobin, and additional investigations should be reviewed according to clinical indications.

#### 3.2.2. Cardiopulmonary Exercise Testing for Functional Assessment and Exercise Prescription

CPET integrates gas exchange, workload, ECG, blood-pressure, and symptom responses to quantify exercise capacity and investigate exercise limitation [4,9]. Peak VO_2_ and the VE/VCO_2_ slope provide prognostic information but require interpretation in relation to the population, clinical context, and test quality. When identifiable, ventilatory thresholds help individualize training intensity; measured heart-rate responses and perceived exertion provide complementary information [9]. Fixed percentages of heart-rate reserve should not be assumed to correspond to the same ventilatory threshold in every patient. CPET supports exercise prescription and reassessment but does not independently establish every HF diagnosis or guarantee exercise safety.

#### 3.2.3. Risk Stratification Algorithms and Contraindications to Exercise Training

Risk assessment integrates clinical stability, symptoms, exercise responses, arrhythmias, ischaemia, and structural disease [4,8]. Decompensated HF during haemodynamic instability, untreated life-threatening arrhythmias, unstable ischaemia, and other major contraindications require treatment before exercise training. NYHA class IV, complex ventricular ectopy, or abnormal exercise blood-pressure responses warrant individualized specialist assessment and an appropriate level of supervision. ECG monitoring should reflect the assessed risk, device characteristics, and rehabilitation setting. Prognostic CPET thresholds should not be treated as universal exercise-exclusion criteria. Appropriate selection, prescription, and monitoring reduce, but do not eliminate, exercise-related risk [18]. Takotsubo syndrome provides a contextual example of acute stress-related cardiac dysfunction that may present with HF and arrhythmic manifestations. A retrospective propensity-matched analysis identified differences in subsequent acute HF and arrhythmic outcomes between patients with Takotsubo syndrome and HF, without a significant difference in all-cause mortality [19]. The study did not evaluate rehabilitation or exercise dose and therefore informs the clinical context rather than supporting a specific exercise protocol.

### 3.3. Tailored Exercise Prescription by Heart Failure Phenotype

In the following subsections, HFrEF, HFmrEF, and HFpEF refer to the historical categories used in the cited literature. Study-specific LVEF eligibility criteria remain relevant when interpreting each result, and the amount of direct rehabilitation evidence differs between these populations.

#### 3.3.1. Heart Failure with Reduced Ejection Fraction (HFrEF)

For stable patients represented in historical HFrEF trials, aerobic training is a core rehabilitation component [4,20]. Frequency, intensity, time, and type should reflect measured exercise capacity, symptoms, haemodynamic responses, comorbidities, and patient goals. A practical framework is gradual progression towards three to five aerobic sessions per week, with duration and intensity adapted to tolerance and supervision determined by risk [4,9]. MICT is an established option, while resistance exercise can complement aerobic training to improve strength and physical function [21]. These functional benefits should be distinguished from the less certain effects on mortality discussed in Section 3.6.

#### 3.3.2. Heart Failure with Preserved Ejection Fraction (HFpEF)

Exercise training in HFpEF should address the individual pattern of exercise limitation rather than assume uniformly severe diastolic stiffness or chronotropic dysfunction [15]. MICT and selected HIIT programmes have been investigated, but OptimEx-Clin did not establish HIIT superiority over MICT for improvement in peak VO_2_ [10]. Neither intervention reached the prespecified clinically important difference compared with guideline-based advice at the primary assessment. Modality and progression should therefore reflect clinical assessment, tolerance, preferences, and feasibility. Improvements in exercise capacity do not necessarily imply normalization of resting diastolic function or reductions in hospitalization and mortality. Diagnostic uncertainty is particularly relevant in older adults, in whom comorbidities and nonspecific findings may complicate attribution of exertional symptoms. A recent letter discusses the possibility of diagnostic expansion in this population; this conceptual concern should be distinguished from primary diagnostic-validation evidence [22].

#### 3.3.3. Heart Failure with LVEF 41–49%—The Historical HFmrEF Category

Historical HFmrEF cohorts, defined by LVEF 41–49%, are heterogeneous and may include patients with different trajectories of ventricular function [12]. Dedicated rehabilitation evidence is limited, so exercise prescription is informed mainly by broader HF evidence and individual clinical assessment. Initial intensity, progression, monitoring, and repeat testing should reflect stability, exercise responses, comorbidities, and changes in clinical status [4,9]. Recommendations in this subgroup should therefore be understood as cautious extrapolation rather than a separately validated HFmrEF-specific training protocol.

#### 3.3.4. Advanced Heart Failure, LVAD, and Post-Transplantation

**Figure 1:**
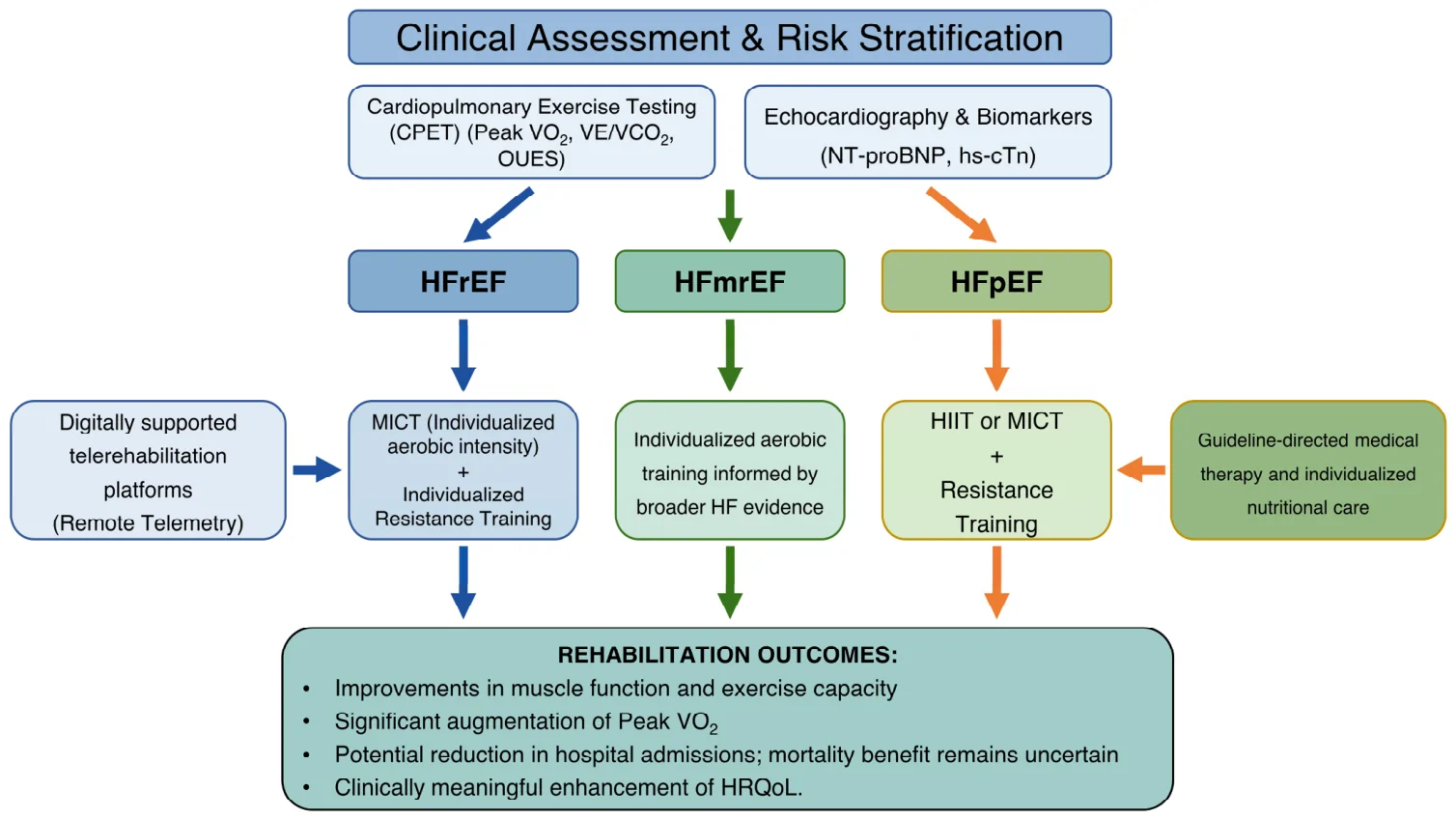
Clinical assessment and individualized rehabilitation across historical LVEF categories. The categories reflect the populations used in the underlying literature and do not represent the revised 2026 ESC classification. Exercise selection, progression, and supervision depend on clinical stability, functional assessment, comorbidities, and patient preferences; the framework is illustrative rather than a validated phenotype-specific algorithm. Abbreviations: CPET, cardiopulmonary exercise testing; HF, heart failure; HFmrEF, heart failure with mildly reduced ejection fraction; HFpEF, heart failure with preserved ejection fraction; HFrEF, heart failure with reduced ejection fraction; HIIT, high-intensity interval training; HRQoL, health-related quality of life; hs-cTn, high-sensitivity cardiac troponin; MICT, moderate-intensity continuous training; NT-proBNP, N-terminal pro-B-type natriuretic peptide; OUES, oxygen uptake efficiency slope; Peak VO_2_, peak oxygen consumption; VE/VCO_2_, minute ventilation to carbon dioxide production.

Rehabilitation in advanced HF, LVAD recipients, and heart transplant recipients requires specialist coordination and consideration of postoperative status, device characteristics, comorbidities, and functional limitations [23,24]. In LVAD recipients, perceived exertion and functional responses can complement device and haemodynamic assessment, because heart rate alone may inadequately represent exercise demand. After transplantation, altered chronotropic responses support individualized intensity assessment and sufficient warm-up and cool-down. Resistance exercise should be introduced progressively according to recovery and surgical precautions [25]. Available evidence, including observational LVAD data, primarily supports improvements in selected functional outcomes, while optimal protocols and effects on major clinical events remain less certain [23,24]. The clinical assessment and exercise-prescription framework is summarized in Figure 1.

### 3.4. Modalities of Physical Rehabilitation

#### 3.4.1. Aerobic Endurance Training: MICT and HIIT Methodologies

Aerobic training can improve exercise capacity in appropriately selected patients with HF [4,20]. MICT is an established approach, while HIIT may be considered in suitable patients according to functional assessment, tolerance, and the available supervision [9]. Selection should account for the patient’s goals, clinical status, and ability to sustain the programme. Comparisons do not establish universal superiority of either modality or equivalence for mortality and hospitalization.

#### 3.4.2. Resistance and Strength Training: Improving Muscle Strength and Physical Function

Resistance training can complement aerobic rehabilitation, particularly when muscle weakness limits daily activities [21]. Load, exercise selection, frequency, and progression should reflect baseline strength, frailty, symptoms, and clinical stability. The clearest supporting evidence concerns improvements in muscle strength and physical function; consistent increases in bone density or reversal of cardiac cachexia should not be assumed [15,21].

#### 3.4.3. Inspiratory Muscle Training: An Adjunct for Selected Patients with Inspiratory Muscle Weakness

Inspiratory muscle training may be considered as an adjunct, particularly when inspiratory muscle weakness contributes to symptoms or limits conventional exercise [26,27]. Resistance and progression should be based on inspiratory muscle assessment and tolerance. Small trials, including TRAINING-HF, support improvements in selected exercise-related outcomes, but these findings do not establish a universal protocol or reductions in hospitalization and mortality [27].

#### 3.4.4. Neuromuscular Electrical Stimulation (NMES): Applications for Severely Deconditioned or Bedbound Patients

NMES may provide an adjunct for selected patients unable to undertake sufficient voluntary exercise. Evidence includes small studies in chronic HF and trials in hospitalized patients with acute HF [28,29]. These findings support selected functional benefits but do not establish unrestricted safety during severe hemodynamic instability. Patient selection, stimulation parameters, and monitoring require clinical assessment. NMES may support progression towards active rehabilitation but does not replace stabilization of decompensated HF.

### 3.5. Multidisciplinary Adjuvants in Cardiac Rehabilitation

#### 3.5.1. Nutritional Interventions: Managing Cardiac Cachexia and Micronutrient Deficiencies

Nutritional assessment should identify unintentional weight loss, inadequate intake, muscle loss, and relevant deficiencies, followed by individualized dietary support [30]. Evidence is insufficient to present specialized branched-chain amino acid supplementation as a routine requirement for all patients undergoing rehabilitation. In SODIUM-HF, dietary sodium reduction did not significantly reduce the primary composite of cardiovascular hospitalization, cardiovascular emergency visits, or all-cause death [31]. Iron deficiency should be evaluated and treated according to applicable HF recommendations and patient eligibility [13]. Intravenous iron is not a universal prerequisite for exercise, and AFFIRM-AHF and HEART-FID should be interpreted according to their specific populations and prespecified endpoints [32,33].

#### 3.5.2. Psychosocial and Behavioral Support: Addressing Depression, Anxiety, and Cognitive Impairment

Psychosocial assessment helps identify depression, anxiety, cognitive difficulties, and barriers to participation in rehabilitation [4,8]. These problems may affect self-care and adherence, but their relationships with HF are multifactorial. Education and behavioral support should be adapted to individual needs, with psychological or cognitive assessment and treatment when indicated. CBT is one potential intervention for appropriate patients rather than a mandatory component for every participant [18]. Evidence concerning psychological outcomes of exercise should be distinguished from evidence evaluating a specific psychological treatment [34].

#### 3.5.3. Pharmacotherapy and Exercise Prescription

Rehabilitation complements guideline-directed medical therapy. Clinical trials and evidence syntheses concerning SGLT2 inhibitors and ARNI provide evidence for drug-specific benefits, but do not by themselves establish synergistic interactions with exercise training [35,36,37,38,39]. Medication-related changes in heart rate, blood pressure, volume status, and symptoms should inform exercise prescription and reassessment. In patients receiving rate-limiting therapy, measured exercise responses and perceived exertion are more informative than reliance on age-predicted maximal heart rate alone [9]. Proposed metabolic mechanisms should be distinguished from demonstrated changes in exercise capacity and clinical outcomes.

### 3.6. Clinical Outcomes and Prognostic Benefits

#### 3.6.1. Impact on All-Cause and HF-Specific Hospitalizations and Mortality

The 2024 Cochrane review included 60 randomized trials involving 8728 participants, predominantly patients with HFrEF. The cited Cochrane review rated the evidence as moderate certainty for short-term all-cause hospital admissions and Minnesota Living with Heart Failure Questionnaire (MLWHFQ) outcomes, and low certainty for short-term all-cause mortality [40]. Compared with usual care, exercise-based cardiac rehabilitation reduced short-term all-cause hospital admissions (RR 0.69, 95% CI 0.56–0.86). The estimate for all-cause mortality did not demonstrate a statistically significant reduction (RR 0.93, 95% CI 0.71–1.21), while the estimate for HF-related admissions was imprecise (RR 0.82, 95% CI 0.49–1.35). These findings support rehabilitation but do not establish a mortality benefit or equivalent effects across LVEF categories. Point estimates suggesting benefit should be interpreted alongside confidence intervals, follow-up duration, and the characteristics of the contributing trials.

#### 3.6.2. Enhancement of Peak VO_2_ and Its Prognostic Significance

Peak VO_2_ provides an objective measure of aerobic exercise capacity and contributes to prognostic assessment in HF. However, an intervention-induced improvement should not automatically be interpreted as evidence of improved survival. TELEREH-HF demonstrated short-term functional improvement without a significant reduction in mortality or hospitalization during subsequent follow-up [41]. Its prognostic subanalysis also illustrates the distinction between identifying patients at different risks and demonstrating a treatment effect [42]. Functional improvement and clinical-event reduction should therefore be reported separately.

#### 3.6.3. Improvements in Health-Related Quality of Life (HRQoL)

HRQoL is an important patient-reported outcome in HF and should be assessed using validated instruments [43]. In the Cochrane review, exercise-based rehabilitation improved short-term Minnesota Living with Heart Failure Questionnaire scores (mean difference −7.39 points, 95% CI −10.30 to −4.77), with lower scores indicating better HRQoL [40]. REACH-HF also demonstrated improvement in disease-specific HRQoL compared with usual care at 12 months [44]. These findings support a patient-centred benefit, while the magnitude and persistence of improvement may vary between programmes and populations.

### 3.7. Limitations of the Study

This review has the limitations of a narrative synthesis. Literature selection and interpretation may be influenced by author judgement, and completeness of retrieval cannot be assured. The review does not provide a formal study-level risk-of-bias assessment or certainty-of-evidence grading. Its conclusions should therefore be interpreted alongside the design, precision, and limitations of the underlying studies.

Comparisons are constrained by differences in patient characteristics, rehabilitation content, exercise intensity, supervision, adherence, comparator care, and follow-up. Functional and patient-reported benefits should not be interpreted as equivalent to reductions in hospitalization or mortality [40]. Applicability to frail patients, those with advanced HF, LVAD recipients, and transplant recipients requires attention to the populations and settings evaluated [17,23,24].

The historical LVEF categories used to organize this review differ from the 2026 ESC classification [13]. Published aggregate results do not necessarily permit reliable reconstruction of outcomes within the revised categories. For example, findings from patients with LVEF ≤ 40% cannot automatically establish the same effect throughout the contemporary HFrEF category of LVEF < 50%. The historical structure is retained to preserve fidelity to the original evidence, while acknowledging that future studies and individual-participant-data analyses may support synthesis aligned with the revised classification. Selected trials and systematic reviews discussed in this narrative review are summarized in Table 1.

### 3.8. Contemporary Challenges and Future Directions

#### 3.8.1. Overcoming Barriers to CR Referral and Adherence

Access to rehabilitation is limited by referral practices, programme availability, travel requirements, financial barriers, and patient-related factors [4,8]. Referral, enrolment, attendance, and programme completion are distinct outcomes and should not be represented by a single global percentage. Service development should address locally identified barriers and offer delivery options compatible with patients’ clinical and practical needs.

#### 3.8.2. Telerehabilitation and Home-Based CR: Efficacy, Safety, and Technological Integration

Home-based and hybrid rehabilitation provide additional options for patients facing barriers to centre-based participation. In TELEREH-HF, short-term functional and quality-of-life improvements did not translate into significant long-term reductions in mortality or hospitalization [41]. Lundgren et al. studied patients declining outpatient rehabilitation and found no significant between-group differences in long-term physical activity, peak VO_2_, or HRQoL [45]. These results should not be interpreted as a non-inferiority comparison against centre-based rehabilitation. Remote monitoring can support supervision, but eligibility, monitoring, and escalation procedures should reflect clinical stability, risk, and patient capabilities.

#### 3.8.3. Precision Medicine in CR: Phenotyping and Multi-Omics Approaches

Multi-omics and artificial intelligence have been proposed as tools for investigating variation in rehabilitation responses and developing individualized strategies [46]. Their clinical application requires prospective validation, assessment of incremental value over standard clinical information, and evaluation of feasibility, cost, and equitable access. These approaches should be presented as research directions rather than established requirements for effective rehabilitation.

## 4. Discussion

The evidence reviewed supports cardiac rehabilitation as a component of HF care, with benefits that are most consistently demonstrated for exercise capacity, physical function, and HRQoL [4,20]. Reductions in all-cause hospital admissions provide additional support, whereas mortality effects remain uncertain [40]. Physiological improvements should therefore be interpreted as valuable outcomes in their own right rather than automatic evidence of improved survival.

The strength and directness of evidence differ between populations. Historical HFrEF cohorts contribute substantially to the evidence base, whereas dedicated data for HFmrEF, advanced HF, LVAD recipients, and transplant recipients are more limited [12,23,24,40]. In HFpEF, heterogeneity of exercise limitation and diagnostic presentation further supports individualized assessment [10,15]. Retaining historical LVEF categories facilitates interpretation of the original studies but does not justify assuming identical effects within contemporary categories.

Clinical implementation requires integration of functional assessment, stability, comorbidities, medication effects, and patient preferences. CPET can inform prescription, but supervision and progression remain clinical decisions rather than consequences of a single physiological threshold [9]. Nutritional and psychosocial interventions should address identified needs, with emerging mechanisms distinguished from established treatment indications.

Home-based and hybrid programmes may broaden access, but their effects depend on programme content, patient selection, and sustained participation [41,44,45]. Future research should evaluate underrepresented populations, maintenance of functional gains, clinical events, and implementation outcomes. Omics-based approaches require validation before they can be recommended for routine exercise prescription [46].

## 5. Conclusions

1. Exercise-based cardiac rehabilitation improves functional capacity and HRQoL in appropriately selected patients with HF and can reduce all-cause hospital admissions. Its effect on mortality remains uncertain, and the strength of evidence differs between populations and outcomes.
2. Exercise prescription should be individualized according to clinical stability, functional assessment, comorbidities, and patient preferences, with nutritional and psychosocial support directed at identified needs.
3. Historical LVEF categories are retained in this review to reflect the populations originally studied.
4. Home-based and hybrid delivery may improve access, whereas omics-guided prescription remains a research direction requiring clinical validation.

## Figures and Tables

**Table 1:** Selected trials and systematic reviews discussed in the narrative review.

No.	Author (Year)	Population	Interventions	Outcomes	Type of HF	Notes
1	Mueller S et al. (2021) [10]	180 randomized patients, 60 per group.	High-Intensity Interval Training (HIIT) vs. Moderate-Intensity Continuous Training (MICT) vs. Guideline Advice.	HIIT did not demonstrate superiority over MICT for peak VO_2_; neither intervention reached the prespecified clinically important difference versus guideline advice at the primary assessment.	HFpEF	Absence of superiority does not establish equivalence.
2	Kitzman DW et al. (2021) [17]	349 older patients hospitalized for acute decompensated HF; 97% were frail or prefrail (REHAB-HF).	Early, transitional, tailored physical rehabilitation (multidomain) vs. usual care.	Improved SPPB score at 3 months compared with usual care: between-group mean difference 1.5 points, 95% CI 0.9–2.0.	HFrEF, HFpEF	Supports early, individualized multidomain rehabilitation to improve physical function over 3 months in selected older patients hospitalized for HF; reductions in rehospitalization or mortality were not established.
3	Ezekowitz JA et al. (2022) [31]	806 patients (SODIUM-HF Trial)	Individualized dietary counseling targeting sodium intake < 1500 mg/day vs. usual care.	No statistically significant reduction in the 12-month composite of cardiovascular hospitalization, cardiovascular emergency-department visits, or all-cause death: HR 0.89, 95% CI 0.63–1.26.	All phenotypes	No demonstrated reduction in the primary clinical composite; patient-reported outcomes should be interpreted separately.
4	Mentz RJ et al. (2023) [33]	3065 patients with iron deficiency (HEART-FID)	Intravenous Ferric carboxymaltose vs. placebo.	No statistically significant benefit at the prespecified 0.01 level for the hierarchical composite of death and HF hospitalizations within 12 months and change in 6MWD at 6 months: unmatched win ratio 1.10, 99% CI 0.99–1.23.	HFrEF	The primary hierarchical outcome was not significantly improved; serious adverse events occurred at similar frequencies in the randomized groups.
5	Packer M et al. (2020) [36]	3730 patients (EMPEROR-Reduced Trial)	Empagliflozin (10 mg) vs. placebo.	Empagliflozin reduced the composite of cardiovascular death or hospitalization for HF compared with placebo: HR 0.75, 95% CI 0.65–0.86.	HFrEF	Pharmacological evidence in patients with LVEF ≤ 40%; the composite result does not independently establish a reduction in cardiovascular mortality or synergy with rehabilitation.
6	Anker SD et al. (2021) [37]	5988 patients (EMPEROR-Preserved Trial)	Empagliflozin (10 mg) vs. placebo.	Empagliflozin reduced the composite of cardiovascular death or HF hospitalization: HR 0.79, 95% CI 0.69–0.90; the effect was mainly related to fewer HF hospitalizations.	HFpEF, HFmrEF	Pharmacological evidence in patients with LVEF > 40%; the trial did not directly evaluate cardiac rehabilitation or drug–exercise synergy.
7	Solomon SD et al. (2022) [38]	6263 patients (DELIVER Trial)	Dapagliflozin (10 mg) vs. placebo.	Dapagliflozin reduced the composite of worsening HF, defined as HF hospitalization or an urgent HF visit, or cardiovascular death: HR 0.82, 95% CI 0.73–0.92, over a median follow-up of 2.3 years.	HFpEF, HFmrEF	Evidence concerns dapagliflozin in patients with LVEF > 40%; the trial did not directly evaluate cardiac rehabilitation or drug–exercise synergy.
8	Molloy C et al. (2024) [40]	8728 participants across 60 randomized trials.	Exercise-based cardiac rehabilitation vs. no exercise control.	Reduced short-term all-cause hospital admissions and improved MLWHFQ scores; mortality and HF-specific admission estimates remained uncertain.	Predominantly HFrEF	Predominantly HFrEF populations; applicability to other groups requires caution.
9	Piotrowicz E et al. (2020) [41]	850 patients (TELEREH-HF Trial)	9-week hybrid comprehensive telerehabilitation vs. usual care.	Short-term functional and HRQoL improvements without a significant long-term benefit in days alive and out of hospital, mortality, or hospitalization.	HFrEF	Safety findings apply to the selected population and monitored programme evaluated.
10	Dalal HM et al. (2019) [44]	216 patients (REACH-HF Trial)	12-week home-based, facilitated CR manual vs. usual care.	Significant and clinically meaningful improvement in the disease-specific Minnesota Living with Heart Failure Questionnaire at 12 months.	HFrEF	Supports a facilitated home-based option for improving disease-specific HRQoL in HFrEF; costs and cost-effectiveness require assessment within the intended healthcare setting.

Abbreviations: 6MWD, 6-min walk distance; CI, confidence interval; CR, cardiac rehabilitation; HF, heart failure; HFmrEF, heart failure with mildly reduced ejection fraction; HFpEF, heart failure with preserved ejection fraction; HFrEF, heart failure with reduced ejection fraction; HIIT, high-intensity interval training; HR, hazard ratio; HRQoL, health-related quality of life; MLWHFQ, Minnesota Living with Heart Failure Questionnaire; MICT, moderate-intensity continuous training; SPPB, short physical performance battery.

## Data Availability

No new data were created or analyzed in this study. Data sharing is not applicable to this article.

## Abbreviations

The following abbreviations are used in this manuscript:

6MWD	6-min walk distance
6MWT	6-min walk test
AI	Artificial intelligence
ARNI	Angiotensin Receptor-Neprilysin Inhibitors
a-vO_2_	Arteriovenous oxygen difference
BNP	B-type natriuretic peptide
CBT	Cognitive-behavioral therapy
CI	Confidence interval
CPET	Cardiopulmonary Exercise Testing
CR	Cardiac rehabilitation
CV	Cardiovascular
ECG	Electrocardiography
ED	Emergency Department
E/e’	Ratio of early diastolic transmitral flow velocity (E) to early diastolic mitral annular velocity (e′)
ESC	European Society of Cardiology
eNOS	Endothelial nitric oxide synthase
ExCR	Exercise-based cardiac rehabilitation
FITT	Frequency, Intensity, Time, Type
GDMT	Guideline-directed medical therapy
HF	Heart failure
HFimpEF	Heart failure with improved ejection fraction
HFmrEF	Heart failure with mildly reduced ejection fraction
HFpEF	Heart failure with preserved ejection fraction
HFrEF	Heart failure with reduced ejection fraction
HIIT	High-intensity interval training
HR	Hazard ratio
HRQoL	Health-Related Quality of Life
HRR	Heart rate reserve
hs-cTn	High-sensitivity cardiac troponin
ICD	Implantable cardioverter-defibrillators
IMT	Inspiratory muscle training
KCCQ	Kansas City Cardiomyopathy Questionnaire
LAVI	Left atrial volume index
LVAD	Left ventricular assist device
LVEF	Left ventricular ejection fraction
MeSH	Medical Subject Headings
MICT	Moderate-intensity continuous training
MLWHFQ	Minnesota Living with Heart Failure Questionnaire
mTOR	Mammalian target of rapamycin
NMES	Neuromuscular electrical stimulation
NT-proBNP	N-terminal pro-B-type natriuretic peptide
NYHA	New York Heart Association
OHT	Orthotopic heart transplant
OUES	Oxygen Uptake Efficiency Slope
Peak VO2	Peak oxygen consumption
PGC-1α	Peroxisome proliferator-activated receptor-gamma coactivator-1 alpha
PI_max_	Maximal inspiratory pressure
RCP	Respiratory compensation point
RCTs	Randomized controlled trials
RPE	Rating of Perceived Exertion
RR	Relative risk
RT	Resistance training
SGLT2(i)	Sodium-glucose cotransporter-2 (inhibitors)
SPPB	Short Physical Performance Battery
TAPSE	Tricuspid annular plane systolic excursion
V/Q	Ventilation–perfusion
VE/VCO_2_	Minute ventilation to carbon dioxide production
VT1	First ventilatory threshold

## References

[1] Savarese G., Becher P.M., Lund L.H., Seferovic P., Rosano G.M.C., Coats A.J.S. (2022). Global burden of heart failure: A comprehensive and updated review of epidemiology. Cardiovasc. Res..

[2] Yan T., Zhu S., Yin X., Xie C., Xue J., Zhu M., Weng F., Zhu S., Xiang B., Zhou X. (2023). Burden, Trends, and Inequalities of Heart Failure Globally, 1990 to 2019: A Secondary Analysis Based on the Global Burden of Disease 2019 Study. J. Am. Heart Assoc..

[3] Groenewegen A., Rutten F.H., Mosterd A., Hoes A.W. (2020). Epidemiology of heart failure. Eur. J. Heart Fail..

[4] Bozkurt B., Fonarow G.C., Goldberg L.R., Guglin M., Josephson R.A., Forman D.E., Lin G., Lindenfeld J., O’Connor C., Panjrath G. (2021). Cardiac Rehabilitation for Patients with Heart Failure: JACC Expert Panel. J. Am. Coll. Cardiol..

[5] Aimo A., Saccaro L.F., Borrelli C., Fabiani I., Gentile F., Passino C., Emdin M., Piepoli M.F., Coats A.J.S., Giannoni A. (2021). The ergoreflex: How the skeletal muscle modulates ventilation and cardiovascular function in health and disease. Eur. J. Heart Fail..

[6] Tucker W.J., Fegers-Wustrow I., Halle M., Haykowsky M.J., Chung E.H., Kovacic J.C. (2022). Exercise for Primary and Secondary Prevention of Cardiovascular Disease: JACC Focus Seminar 1/4. J. Am. Coll. Cardiol..

[7] McDonagh T.A., Metra M., Adamo M., Gardner R.S., Baumbach A., Böhm M., Burri H., Butler J., Čelutkienė J., Chioncel O. (2021). 2021 ESC Guidelines for the diagnosis and treatment of acute and chronic heart failure. Eur. Heart J..

[8] Ambrosetti M., Abreu A., Corrà U., Davos C.H., Hansen D., Frederix I., Iliou M.C., Pedretti R.F.E., Schmid J.P., Vigorito C. (2021). Secondary prevention through comprehensive cardiovascular rehabilitation: From knowledge to implementation. 2020 update. A position paper from the Secondary Prevention and Rehabilitation Section of the European Association of Preventive Cardiology. Eur. J. Prev. Cardiol..

[9] Hansen D., Abreu A., Ambrosetti M., Cornelissen V., Gevaert A., Kemps H., Laukkanen J.A., Pedretti R., Simonenko M., Wilhelm M. (2022). Exercise intensity assessment and prescription in cardiovascular rehabilitation and beyond: Why and how: A position statement from the Secondary Prevention and Rehabilitation Section of the European Association of Preventive Cardiology. Eur. J. Prev. Cardiol..

[10] Mueller S., Winzer E.B., Duvinage A., Gevaert A.B., Edelmann F., Haller B., Pieske-Kraigher E., Beckers P., Bobenko A., Hommel J. (2021). Effect of High-Intensity Interval Training, Moderate Continuous Training, or Guideline-Based Physical Activity Advice on Peak Oxygen Consumption in Patients with Heart Failure with Preserved Ejection Fraction: A Randomized Clinical Trial. JAMA.

[11] Heidenreich P.A., Bozkurt B., Aguilar D., Allen L.A., Byun J.J., Colvin M.M., Deswal A., Drazner M.H., Dunlay S.M., Evers L.R. (2022). 2022 AHA/ACC/HFSA Guideline for the Management of Heart Failure: A Report of the American College of Cardiology/American Heart Association Joint Committee on Clinical Practice Guidelines. Circulation.

[12] Savarese G., Stolfo D., Sinagra G., Lund L.H. (2022). Heart failure with mid-range or mildly reduced ejection fraction. Nat. Rev. Cardiol..

[13] Køber L., Adamo M., Ruwald A.-C., Tomasoni D., Anderson L.J., Andersson C., Brugts J.J., Chioncel O., Donal E., Ferdinandy P. (2026). 2026 ESC Guidelines for the management of heart failure. Eur. Heart J..

[14] Nair N. (2021). Invasive Hemodynamics in Heart Failure with Preserved Ejection Fraction: Importance of Detecting Pulmonary Vascular Remodeling and Right Heart Function. Heart Fail. Clin..

[15] Mirzai S., Sandesara U., Haykowsky M.J., Brubaker P.H., Kitzman D.W., Peters A.E. (2025). Aerobic, resistance, and specialized exercise training in heart failure with preserved ejection fraction: A state-of-the-art review. Heart Fail. Rev..

[16] Nagueh S.F., Sanborn D.Y., Oh J.K., Anderson B., Billick K., Derumeaux G., Klein A., Koulogiannis K., Mitchell C., Shah A. (2025). Recommendations for the Evaluation of Left Ventricular Diastolic Function by Echocardiography and for Heart Failure with Preserved Ejection Fraction Diagnosis: An Update from the American Society of Echocardiography. J. Am. Soc. Echocardiogr..

[17] Kitzman D.W., Whellan D.J., Duncan P., Pastva A.M., Mentz R.J., Reeves G.R., Nelson M.B., Chen H., Upadhya B., Reed S.D. (2021). Physical Rehabilitation for Older Patients Hospitalized for Heart Failure. N. Engl. J. Med..

[18] Bäck M., Wilhelm M., Marcin T., Machado F.V.C., Sandberg A., Buccheri S., Castro Conde A., Czapla M., De Rosa S., Gibson I. (2026). 2026 ESC Guidelines on cardiac rehabilitation. Eur. Heart J..

[19] Tartaglia E., Alobaida M., Bucci T., Rossi M., Askarinejad A., Lam H.M., Kaskal M., Rigutini A.G., Boriani G., Lip G.Y.H. (2026). Long-term prognosis in Takotsubo syndrome compared to heart failure: Observations from a global federated research network. ESC Heart Fail..

[20] Taylor R.S., Dalal H.M., McDonagh S.T.J. (2022). The role of cardiac rehabilitation in improving cardiovascular outcomes. Nat. Rev. Cardiol..

[21] Fisher S., Smart N.A., Pearson M.J. (2022). Resistance training in heart failure patients: A systematic review and meta-analysis. Heart Fail. Rev..

[22] Bellicini M.G. (2026). HFpEF in older patients: Diagnostic uncertainty or diagnostic expansion?. Am. J. Med..

[23] Schlöglhofer T., Gross C., Moscato F., Neumayer A., Kandioler E., Leithner D., Skoumal M., Laufer G., Wiedemann D., Schima H. (2023). Exercise Performance and Quality of Life of Left Ventricular Assist Device Patients After Long-Term Outpatient Cardiac Rehabilitation. J. Cardiopulm. Rehabil. Prev..

[24] Costa R., Moreira E., Silva Cardoso J., Azevedo L.F., Ribeiro J.A., Pinto R. (2023). Effectiveness of Exercise-Based Cardiac Rehabilitation for Heart Transplant Recipients: A Systematic Review and Meta-Analysis. Health Serv. Insights.

[25] Simonenko M., Hansen D., Niebauer J., Volterrani M., Adamopoulos S., Amarelli C., Ambrosetti M., Anker S.D., Bayes-Genis A., Ben Gal T. (2024). Prevention and rehabilitation after heart transplantation: A clinical consensus statement of the European Association of Preventive Cardiology, Heart Failure Association of the ESC, and the European Cardio Thoracic Transplant Association, a section of ESOT. Eur. J. Prev. Cardiol..

[26] Isakoglou M., Kortianou E.A. (2026). Inspiratory Muscle Training in Heart Failure as a Promising Tool in the Heart Failure Toolkit: From Physiology to Practice. Encyclopedia.

[27] Palau P., Domínguez E., López L., Ramón J.M., Heredia R., González J., Santas E., Bodí V., Miñana G., Valero E. (2019). Inspiratory Muscle Training and Functional Electrical Stimulation for Treatment of Heart Failure with Preserved Ejection Fraction: The TRAINING-HF Trial. Rev. Esp. Cardiol. (Engl. Ed.).

[28] Ono S., Kato M., Seko H., Nakatani E., Omote T., Omote M., Omote S. (2025). Randomised Crossover Trial of Home-Based Neuromuscular Electrical Stimulation Therapy as an Adjunct to Cardiac Rehabilitation in Frail Older Adult Patients with Chronic Heart Failure. Circ. Rep..

[29] Yang F.A., Hong Y.H., Tseng Y.L., Harnod D., Chen H.C. (2026). A systematic review and meta-analysis of randomized controlled trials on the effects of neuromuscular electrical stimulation in patients with acute heart failure. PeerJ.

[30] Bianchi V.E. (2020). Nutrition in chronic heart failure patients: A systematic review. Heart Fail. Rev..

[31] Ezekowitz J.A., Colin-Ramirez E., Ross H., Escobedo J., Macdonald P., Troughton R., Saldarriaga C., Alemayehu W., McAlister F.A., Arcand J. (2022). Reduction of dietary sodium to less than 100 mmol in heart failure (SODIUM-HF): An international, open-label, randomised, controlled trial. Lancet.

[32] Ponikowski P., Kirwan B.A., Anker S.D., McDonagh T., Dorobantu M., Drozdz J., Fabien V., Filippatos G., Göhring U.M., Keren A. (2020). Ferric carboxymaltose for iron deficiency at discharge after acute heart failure: A multicentre, double-blind, randomised, controlled trial. Lancet.

[33] Mentz R.J., Garg J., Rockhold F.W., Butler J., De Pasquale C.G., Ezekowitz J.A., Lewis G.D., O’Meara E., Ponikowski P., Troughton R.W. (2023). Ferric Carboxymaltose in Heart Failure with Iron Deficiency. N. Engl. J. Med..

[34] Wang Y.C., Apriliyasari R.W., Jeng C., Hsieh M.H., Tsai P.S. (2025). Effects of Home-Based Exercise on Depression, Anxiety, Functional Capacity, and Quality of Life in Patients with Heart Failure with Reduced Ejection Fraction: A Systematic Review and Meta-analysis of Randomized Controlled Trials. J. Cardiovasc. Nurs..

[35] Santos-Gallego C.G., Vargas-Delgado A.P., Requena-Ibanez J.A., Garcia-Ropero A., Mancini D., Pinney S., Macaluso F., Sartori S., Roque M., Sabatel-Perez F. (2021). Randomized Trial of Empagliflozin in Nondiabetic Patients with Heart Failure and Reduced Ejection Fraction. J. Am. Coll. Cardiol..

[36] Packer M., Anker S.D., Butler J., Filippatos G., Pocock S.J., Carson P., Januzzi J., Verma S., Tsutsui H., Brueckmann M. (2020). Cardiovascular and Renal Outcomes with Empagliflozin in Heart Failure. N. Engl. J. Med..

[37] Anker S.D., Butler J., Filippatos G., Ferreira J.P., Bocchi E., Böhm M., Brunner-La Rocca H.-P., Choi D.-J., Chopra V., Chuquiure-Valenzuela E. (2021). Empagliflozin in Heart Failure with a Preserved Ejection Fraction. N. Engl. J. Med..

[38] Solomon S.D., McMurray J.J.V., Claggett B., de Boer R.A., DeMets D., Hernandez A.F., Inzucchi S.E., Kosiborod M.N., Lam C.S.P., Martinez F. (2022). Dapagliflozin in Heart Failure with Mildly Reduced or Preserved Ejection Fraction. N. Engl. J. Med..

[39] Bhattacharjee P., Khan Z. (2023). Sacubitril/Valsartan in the Treatment of Heart Failure with Reduced Ejection Fraction Focusing on the Impact on the Quality of Life: A Systematic Review and Meta-Analysis of Randomized Clinical Trials. Cureus.

[40] Molloy C., Long L., Mordi I.R., Bridges C., Sagar V.A., Davies E.J., Coats A.J.S., Dalal H., Rees K., Singh S.J. (2024). Exercise-based cardiac rehabilitation for adults with heart failure. Cochrane Database Syst. Rev..

[41] Piotrowicz E., Pencina M.J., Opolski G., Zaręba W., Banach M., Kowalik I., Orzechowski P., Szalewska D., Pluta S., Główczyńska R. (2020). Effects of a 9-Week Hybrid Comprehensive Telerehabilitation Program on Long-term Outcomes in Patients with Heart Failure: The Telerehabilitation in Heart Failure Patients (TELEREH-HF) Randomized Clinical Trial. JAMA Cardiol..

[42] Piotrowicz E., Pencina M., Kowalik I., Orzechowski P., Banach M., Glowczynska R., Zareba W., Opolski G., Szalewska D., Pluta S. (2023). Predictors of long-term prognosis based on clinical status and measurements obtained in heart failure patients after 9-week hybrid comprehensive telerehabilitation: A subanalysis of the TELEREH-HF randomized clinical trial. Kardiol. Pol..

[43] Major K., Bodys-Pełka A., Grabowski M., Lelonek M. (2024). Quality of life in heart failure: New data, new drugs and devices. Cardiol. J..

[44] Dalal H.M., Taylor R.S., Jolly K., Davis R.C., Doherty P., Miles J., van Lingen R., Warren F.C., Green C., Wingham J. (2019). The effects and costs of home-based rehabilitation for heart failure with reduced ejection fraction: The REACH-HF multicentre randomized controlled trial. Eur. J. Prev. Cardiol..

[45] Lundgren K.M., Langlo K.A.R., Salvesen Ø., Aspvik N.P., Mo R., Ellingsen Ø., Vesterbekkmo E., Zanaboni P., Dalen H., Aksetøy I.A. (2025). Exercise-Based Telerehabilitation for Heart Failure Patients Declining Outpatient Rehabilitation-A Randomized Controlled Trial. Med. Sci. Sports Exerc..

[46] Ghram A., Lavie C.J. (2025). Integrating omics to optimize precision cardiac rehabilitation. Metab. Target Organ Damage.

